# Optimization and regeneration kinetics of lymphatic-specific photodynamic therapy in the mouse dermis

**DOI:** 10.1007/s10456-013-9365-6

**Published:** 2013-07-28

**Authors:** Witold W. Kilarski, Angelika Muchowicz, Malgorzata Wachowska, Renata Mężyk-Kopeć, Jakub Golab, Melody A. Swartz, Patrycja Nowak-Sliwinska

**Affiliations:** 1 Institute of Bioengineering and Swiss Institute for Cancer Research (ISREC), School of Life Sciences, SV-IBI-LLCB, Station 15, Ecole Polytechnique Fédérale de Lausanne (EPFL), 1015 Lausanne, Switzerland; 2 Department of Immunology, Center of Biostructure Research, Medical University of Warsaw, Warsaw, Poland; 3 Faculty of Biochemistry, Biophysics, and Biotechnology, Jagiellonian University, Kraków, Poland; 4 Department of Urology, CHUV University Hospital, Rue du Bugnon 46, 1011 Lausanne, Switzerland

**Keywords:** Endothelium, Lymphatic collectors, Intravital imaging, Lymphangiogenesis, Lymphangiography, Lymphatic ablation, Reactive oxygen species, Verteporfin, Visudyne, PDT

## Abstract

Lymphatic vessels transport fluid, antigens, and immune cells to the lymph nodes to orchestrate adaptive immunity and maintain peripheral tolerance. Lymphangiogenesis has been associated with inflammation, cancer metastasis, autoimmunity, tolerance and transplant rejection, and thus, targeted lymphatic ablation is a potential therapeutic strategy for treating or preventing such events. Here we define conditions that lead to specific and local closure of the lymphatic vasculature using photodynamic therapy (PDT). Lymphatic-specific PDT was performed by irradiation of the photosensitizer verteporfin that effectively accumulates within collecting lymphatic vessels after local intradermal injection. We found that anti-lymphatic PDT induced necrosis of endothelial cells and pericytes, which preceded the functional occlusion of lymphatic collectors. This was specific to lymphatic vessels at low verteporfin dose, while higher doses also affected local blood vessels. In contrast, light dose (fluence) did not affect blood vessel perfusion, but did affect regeneration time of occluded lymphatic vessels. Lymphatic vessels eventually regenerated by recanalization of blocked collectors, with a characteristic hyperplasia of peri-lymphatic smooth muscle cells. The restoration of lymphatic function occurred with minimal remodeling of non-lymphatic tissue. Thus, anti-lymphatic PDT allows control of lymphatic ablation and regeneration by alteration of light fluence and photosensitizer dose.

**Electronic supplementary material:**

The online version of this article (doi:10.1007/s10456-013-9365-6) contains supplementary material, which is available to authorized users.

## Introduction

Lymphatic vessels drain fluid, antigens, and immune cells from the interstitial space to lymph nodes (LNs) and eventually back into the systemic blood circulation [1]. Initial lymphatic capillaries consist of a monolayer of lymphatic endothelial cells (LECs) attached to a thin basement membrane, and unlike larger lymphatic vessels, they are deprived of smooth muscle cells (SMCs). They collect interstitial fluid, forming lymph, and drain to the pre-collecting and then collecting lymphatic vessels that are surrounded by SMCs and segmented with bicuspid valves. This physiological process regulates the tissue fluid homeostasis, leukocyte recirculation, and transport of antigen-presenting cells to the lymph nodes, crucial for adaptive immunity [2, 3]. Post-developmental lymphangiogenesis occurs primarily during inflammation [4, 5], both in the inflamed tissue as well as its draining lymph node, and in lymph nodes following infection and vaccination [6, 7] or after tissue transplantation [8]. Although its precise immunological functions remain unclear [4, 9], inflammatory lymphangiogenesis has been associated with both immune tolerance as well as immunogenicity. In melanoma, tumor-driven lymphangiogenesis promoted tolerance induction by suppression and deletion of tumor-reactive T cells [10], and lymphangiogenesis has been shown to help resolve inflammation after lung injury [11]. However, corneal lymphangiogenesis is a major risk factor for corneal transplant rejection [12–15], and blocking lymphangiogenesis after experimental islet transplantation helped prevent rejection [16]. VEGF-C or VEGF-D-driven lymphangiogenesis can be prevented by function-blocking antibodies against the lymphatic receptor VEGFR-3 [17–19]. However, anti-lymphangiogenic treatment has no influence on the function of pre-existing lymphatic vessels. Thus, there is great potential, for both fundamental lymphatic research as well as immunotherapeutic strategies, for methods that can locally destroy lymphatic vessels, and particularly lymphatic collectors.

Photodynamic therapy (PDT) is a clinical treatment for ablating unwanted or pathological blood vessels based on the administration of a non-toxic photosensitizer followed by sub-thermal light exposure that induces photosensitizer toxicity via generation of reactive oxygen species (ROS), resulting in thrombosis and vascular occlusion [20–23]. This strategy can be used to treat cancer, as well as non-oncological conditions, such as age-related macular degeneration (AMD) [24] or polypoidal choroidal vasculopathy [25]. Visudyne^®^, a liposomal formulation of verteporfin (the benzoporphyrin derivative monoacid ring A), is a clinically used photosensitizer administrated intravenously in patients for photodynamic treatment of pathologic cornea neovascularization [26, 27] AMD [28] or polypoidal choroidal vasculopathy [25]. Light-activated verteporfin induces a cascade of reactions leading to the formation of highly reactive and toxic ROS, mainly singlet oxygen [29, 30]. Although the effects of PDT on the blood vessels have been extensively studied during the past several decades [20, 23] the use of PDT for lymphatic ablation has only begun to be explored, namely for targeting peritumoral lymphatics and the in-transit tumor cells they contained to treat metastatic disease [31].

Our goal was to determine whether lymphatic vessels could be specifically targeted in skin, and to identify the optimal conditions to selectively close lymphatic collecting vessels without injuring the blood vasculature. More specifically, we focused on (1) the timing and the mechanism of PDT-induced lymphatic-specific closure using optimal light fluencies and photosensitizer doses, and (2) the kinetics of ensuing lymphatic regeneration. Such information, although presumably specific to mouse dermal lymphatics, is necessary to further develop lymphatic-specific PDT, for example to control metastatic spread or perform basic lymphatic research.

## Materials and methods

### PDT on the mouse ear dermis

**Fig. 1:**
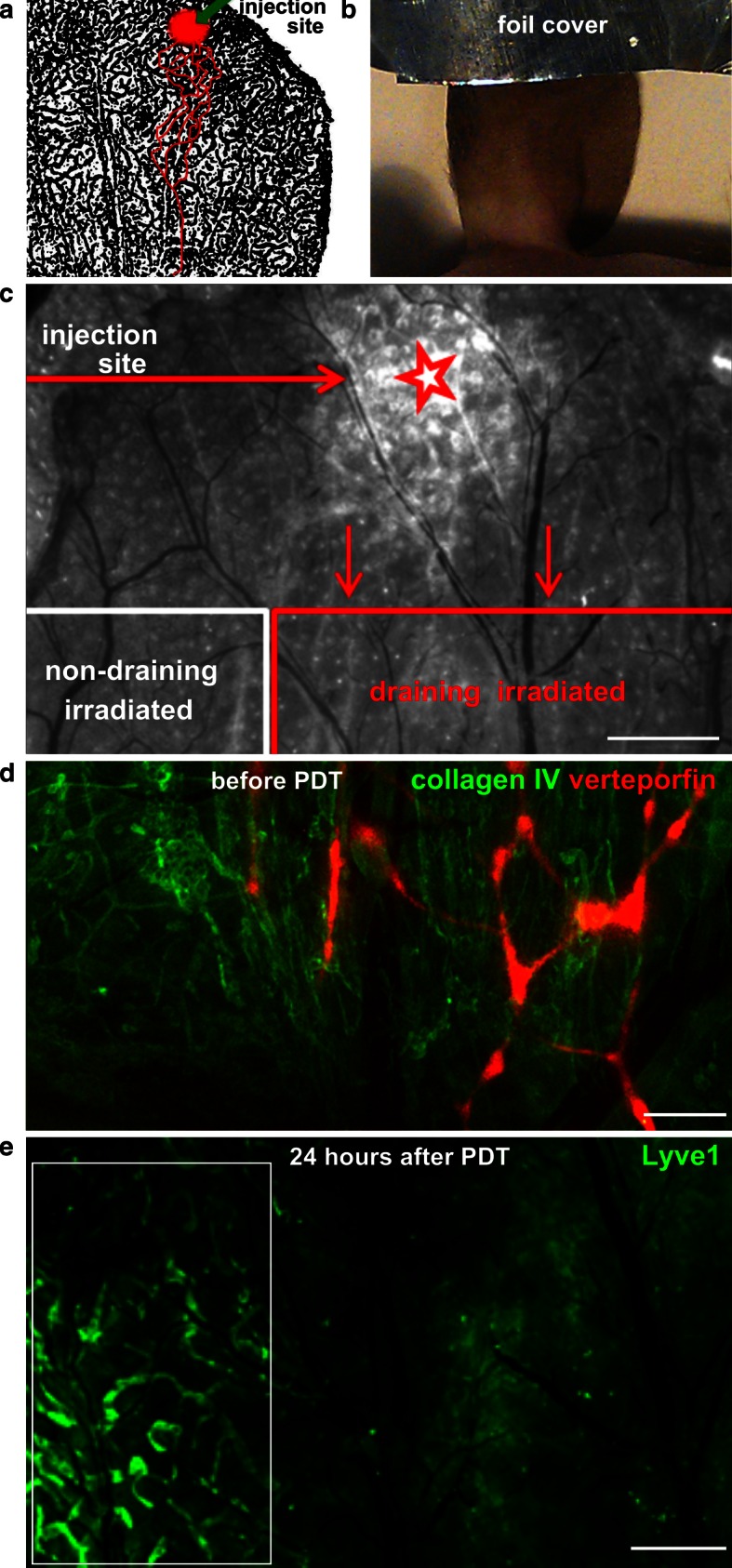
Lymphatic-specific ablation by verteporfin-PDT in the mouse ear. **a** Schematic representation of the injection site and draining lymphatic vessels against a LYVE-1-stained image of the mouse ear lymphatic vasculature (*black*). (*red circle*). **b** The site of verteporfin injection was covered with foil and the rest of the ear was irradiated. **c** Superimposition of verteporfin fluorescence over the bright field image of the exposed dorsal ear dermis 24 h after PDT. Image shows the site of verteporfin injection (*star*) and the verteporfin-draining and non-draining (control) regions in the dorsal ear dermis after removal of ventral skin and intermediate cartilage. *Star* depicts the site of verteporfin injection. *Bar* 0.5 mm *Red star* depicts the site of verteporfin injection (as in **a**). **d** Fluorescence image showing verteporfin (*red*) draining within lymphatic vessels in the exposed dorsal skin after immunostaining for collagen IV (*green*). **e** Destruction of lymphatic endothelium is seen 24 h after PDT by intravital immunofluorescence for LYVE-1. Intact lymphatic vessels are seen in the control (non-draining) region (*white box*). *Scale bar* correspond to 500 μm

All experiments were carried out in BALB/c mice according to a protocol approved by the Committee for Animal Experiments for the Canton Vaud, Switzerland (permits 2316 and 2464). Three days before each experiment, ears were treated with depilation cream (Veet^®^ Hair Removal Cream, Slough, UK) for 10 s followed by thorough rinsing with water. For PDT, mice were anesthetized with 2.5 % isofluorane (Rothhacher GmbH, Bern, Switzerland) and kept under 1.5 % isofluorane for the duration of the experiment. Mouse core temperature was maintained at 37 °C throughout the experiment (DC Temperature Control System, FHC Inc., Bowdoin, MA). A liposomal formulation of verteporfin (Visudyne^®^, Novartis Ophthalmics, Hettlingen, Switzerland) was freshly reconstituted from powder as previously described [32] and injected (0.5 μl) into the tip of the ear dermis using a microsyringe (Hamilton, Reno, NV, USA) (Fig. 1a). The injection site was covered with an aluminum foil (Fig. 1b) and after 5 min, the entire ear was irradiated (Fig. 1c) with non-thermal laser light (Biolitec AG, Jena, Germany) at 689 ± 2 nm delivered through an optical fiber containing a frontal light distributor with lens (Medlight SA, Ecublens, Switzerland) with the laser power of 0.1–1.56 W, and light fluencies between 0.4 and 25 J/cm^2^. Light fluencies were adjusted with neutral density filters and measured with a calibrated Field-Master GS power meter (Coherent Inc, Santa Clara, CA, USA).

### Fluorescence microlymphangiography

In order to identify the effective verteporfin dose necessary to ablate lymphatic vessels selectively, 25 or 100 ng of verteporfin was injected (in 0.5 μl) into the tip of the ear 5 min prior to PDT and ears were imaged with an automated fluorescence stereomicroscope (M205 FA, Leica Microsystems GmbH, Wetzlar, Germany) equipped with a 647 nm filter and camera (DFC350 FX, Leica), controlled by a Leica AF software. Two hours after PDT, 0.5 μl TRITC-dextran (150 kDa; 10 mg/ml; Sigma-Aldrich) was injected in the tip of the ear, near the location of the verteporfin injection. Draining collecting lymphatics were imaged using fluorescence stereomicroscopy with a 594 nm filter (Leica). This was repeated 24 h later to verify the effectiveness of anti-lymphatic PDT.

### Fluorescence angiography

To identify functional, blocked, or leaky blood vessels in the ear, 200 μl TRITC-dextran was injected into the tail vein 24 h after PDT, and the ears were imaged at low magnification. To observe blood perfusion at the level of individual blood vessels, the dorsal and ventral skin of the ear were separated 24 h after PDT, the tail was injected with 200 μl TRITC-dextran, and exposed dorsal circulation in the ear was imaged at high magnification.

### Intravital immunofluorescence lymphangiography

This method is based on the observation that intravital immunolabeling of tissue structures is dependent on intradermal fluid currents (i.e., functional lymphatic drainage) and allows detection of dysfunctional skin drainage basins in the whole explanted tissue despite the presence of intermediate functional lymphatics [33]. Briefly, 24 h after PDT, the ventral skin with underlying cartilage was separated and removed from the dorsal skin, leaving it innervated and functionally connected to the mouse circulation. Then, the tissue was incubated with rabbit anti-Lyve-1 antibody (1 μg/ml, ReliaTech GmbH, Wolfenbüttel, Germany) 15 min, followed by washing in Ringer’s buffer (102 mM NaCl, 5 mM KCl, 2 mM CaCl_2_, 28 mM sodium lactate, 25 mM HEPES), 15 min incubation with Alexa 488 anti-rabbit antibody (1 μg/ml, Invitrogen, Carlsbad, CA, USA). Within this short incubation time, only functionally draining lymphatic vessels became brightly stained for Lyve-1, since convective flows carried the antibodies to draining lymphatic vessels, where they concentrated. In contrast, areas that lacked functional lymphatic drainage had weaker staining because diffusive transport was less significant over this short time period.

After bathing the skin in ascorbate-Ringer solution (140 mM sodium ascorbate, 25 mM HEPES, 4 mM KCl and 2 mM CaCl_2_, at a pH of 7.5) to prevent phototoxicity [33], a coverslip was placed over the ear and lymphatic capillaries were imaged using fluorescence microscopy as described above.

### Intravital immunofluorescence of lymphatic vessels

Immunostaining on the live ear tissue was performed with modifications as described [33]. To detect necrotic cells within lymphatic vessels before PDT, the middle part of the ventral ear skin and cartilage were separated and removed from the dorsal skin, leaving a “dorsal dermis window”. Fcγ receptor blocking solution was placed on the tissue, followed by 15 min incubation with biotinylated rabbit anti-collagen IV (1 μg/ml; Abcam, Cambridge, MA USA), a brief rinse with Ringer’s solution and 15 min incubation with streptavidin-Pacific Blue (1 μg/ml; Invitrogen, Zug, Switzerland). PDT was then performed as described above; the dorsal dermis was mounted under a coverslip in ascorbate-Ringer solution supplemented with propidium iodide (1 μg/ml; Ebioscience, San Diego, CA USA) and imaged for 3 h with the stereomicroscopy setup described above.

To image both the initial and collecting lymphatic vessels, the entire ventral skin and cartilage of the ear was removed and the dorsal dermis was incubated in Fcγ-receptor blocking solution followed by rabbit anti-Lyve-1 and goat anti-podoplanin (R&D Systems, Minneapolis, MN) antibodies for 15 min. After washing with Ringer’s buffer, Alexafluor-labeled secondary antibodies (1 μg/ml, Invitrogen) were applied for another 15 min. Following another wash and 15 min blocking with 20 % goat serum, the ear was incubated in biotinylated goat anti-perlecan antibody (1 μg/ml; Abcam), followed by rinsing and 15 min incubation of fluorescently labeled streptavidin (1 μg/ml). The stained lymphatic structures were then imaged as above.

### Time of lymphatic occlusion and regeneration

PDT was performed with an optimal dose of verteporfin and light fluence of either 3.6 or 25 J/cm^2^. Fluorescence microlymphangiography (above) was performed immediately after PDT as well as at various time points thereafter. After each injection, ears were washed with Betadine solution (MundiPharma) and animals were returned to cages. Lymphatic function was considered “recovered” when the lymphatic drainage was observed in at least one continuous lymphatic collecting vessel spanning the entire ear.

For time of lymphatic occlusion, PDT was performed with an optimal dose of verteporfin and two light doses 25 and 3.6 J/cm^2^. To test lymphatic perfusion fluorescence lymphangiography was performed with TRITC-dextran injected 3, 6, 9 and 24 h post PDT under isoflurane anesthesia.

### Whole-mount staining of the ear dermis

Mice were sacrificed with CO_2_ and exsanguinated by intracardiac perfusion with 20 ml Ringer’s buffer (supplemented with 10,000 IU/l heparin, 0.1 % glucose, 0.1 % procaine and 25 mM HEPES (Sigma-Aldrich); pH 7.5, 330 mOsM) at a constant gravitational pressure of 120 mm Hg. Ringer’s buffer was then replaced to osmolarity-corrected zinc fixative (Zn-fix) solution (4.5 mM CaCl_2_, 52 mM ZnCl_2_, 32 mM Zn(CF_3_COO)_2_, 2 mM Tris, 38 mM glycine; pH 6.5, 340 mOsm/l) [34]. Subsequently, the ears were cut and placed in ice-cold Zn-fix with 1 % Triton^®^ X-100 (Fluka) for at least 24 h. After that, the ventral part of the skin was removed together with cartilage and muscle, and the dorsal dermis was washed in TBS (140 mM NaCl, 25 mM Tris, pH 7.5) for 6 h, blocked with 0.5 % casein in TBS (blocking solution) for 2 h, and incubated for 24 h with the following primary antibodies (1 μg/ml): VE-cadherin, anti-Lyve1, biotinylated anti-collagen type IV, anti-α smooth muscle actin (1A4) (Abcam, Cambridge, UK), biotinylated anti-CD45 (BD Biosciences), biotinylated anti-perlecan (Life Technologies), anti-CD31 (GeneTex) or anti-podoplanin (R&D Systems). After washing in TBS with 0.1 % Tween^®^ 20 (Sigma-Aldrich), the tissue was incubated with 1 μg/ml of each of the appropriate secondary antibodies (all from Abcam or Invitrogen) for another 24 h, subsequently washed in TBS and dehydrated with 70 and 100 % ethanol. Thereafter, the sample was cleared with 2:1 benzyl benzoate/benzyl alcohol solution (refractive index 1.56) supplemented with 25 mg/ml propyl gallate The tissue was mounted on a glass slide and imaged using HC PL APO 20x, NA 0.70 or HCX PL APO 63x, NA 1.40 lenses and a confocal microscope (Leica SP5). Images were analyzed with Imaris 7.1 (Bitplane AG, Zürich, Switzerland).

### Statistical analysis

The effect of verteporfin dose on blood vessel leakage was tested using Fisher’s exact test. Survival curves were compared using the Mantel-Cox test using Prism software (Graphpad, San Diego, CA). Figures were assembled in Cytosketch (Cytokode, Auckland, New Zeland). Results were considered significant when *p* < 0.05.

## Results

### Lymphatic ablation with PDT in the mouse ear dermis

After injecting 100 ng Verteporfin (0.5 μl of 200 μg/ml solution) into the dorsal ear skin as described above (Fig. 1a, b), the naturally fluorescent verteporfin was observed in the draining lymphatic vessels within 2–4 min after injection. Since only a few lymphatic vessels drained the injection area, subsequent irradiation of the entire ear 5 min after injection, with a constant light dose of 3.6 J/cm^2^ produced both a treated region (irradiated, verteporfin-draining) as well as internal control region (irradiated, non-Verteporfin draining) (Fig. 1c). This treatment led to occlusion of the lymphatic collecting vessels in the verteporfin-draining area, but not in the control areas, as shown by intravital immunofluorescence lymphangiography (Fig. 1d, e), which detects the functional drainage basins in the whole tissue [33]. Lymphatic vessels in the areas that did not drain the photosensitizer (box, Fig. 1e) remained unaffected and served as internal controls in the imaging experiments.

### Effects of photosensitizer dose and light fluence on lymphatic ablation

**Fig. 2:**
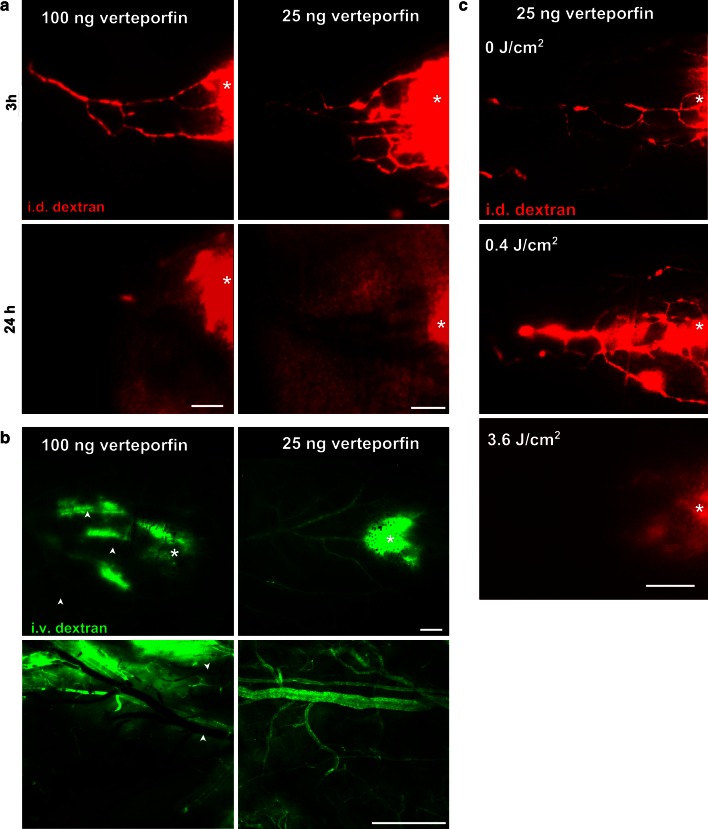
Lymphatic-specific targeting by PDT can be achieved with low doses of intradermally injected photosensitizer. **a** Fluorescence microlymphangiography 3 and 24 h post-PDT shows blockage of lymphatic drainage with both 100 ng (*left*) or 25 ng (*right*) of verteporfin. **b** Fluorescence angiography 24 h after PDT. Blood vessel integrity as revealed by intravascular injection of FITC-dextran (*green*) 24 h post-PDT shows vascular blockage with 100 ng verteporfin (*left*) in all 7 ears tested but no apparent vascular blockage with 25 ng verteporfin (*right*) in 6 out of 7 tested ears (*p* = 0.0047, Fisher exact test). In any case, dextran leaked from blood vessel at the sites of verteporfin injection (*star*). *Top panel* fluorescence angiography in dorsal ear skin imaged through the intact ear. *Bottom panel* high resolution fluorescence angiography in dorsal ear after surgical resection of ventral skin and cartilage. **c** Lymphatic drainage function 24 h after PDT (with 25 ng verteporfin) by fluorescence microlymphangiography with TRITC-dextran (*red*) shows that a light fluence of 3.6 J/cm^2^ but not 0.4 J/cm^2^ could occlude lymphatic drainage in the ear. *Bar* 500 μm

Our first aim was to determine the PDT conditions, including doses of verteporfin and light that allowed for effective and specific closure of lymphatic vasculature. With a fixed light fluence of 25 J/cm^2^, we found that the lowest dose of verteporfin (5 ng) did not cause any visible lymphatic damage (data not shown), while both 25 and 100 ng verteporfin led to lymphatic occlusion, as seen using fluorescence microlymphangiography 24 h post-PDT (Fig. 2a). However, intradermal administration of the high dose (100 ng) of verteporfin caused blood vessel damage as well, as seen by leakage and occlusion of blood vessels at the irradiation site (Fig. 2b, left). In contrast, blood vessels maintained patency when PDT was performed with 25 ng verteporfin, with (Fig. 2b right).

Once the lymphatic-specific dose of verteporfin was identified, we then determined the minimal light fluence required to destroy the lymphatics. As visualized 24 h after PDT with fluorescence microlymphangiography, a light fluence of 0.4 J/cm^2^ did not induce lymphatic ablation, but only increased the permeability of lymphatic vasculature, leading to visible dextran leakage (Fig. 2c). However, PDT with light doses of 3.6 J/cm^2^ and above led to a complete occlusion of the lymphatic vessels without affecting blood vessel permeability (Fig. 2b).

### Lymphatic occlusion is delayed compared to lymphatic endothelial cell death

**Fig. 3:**
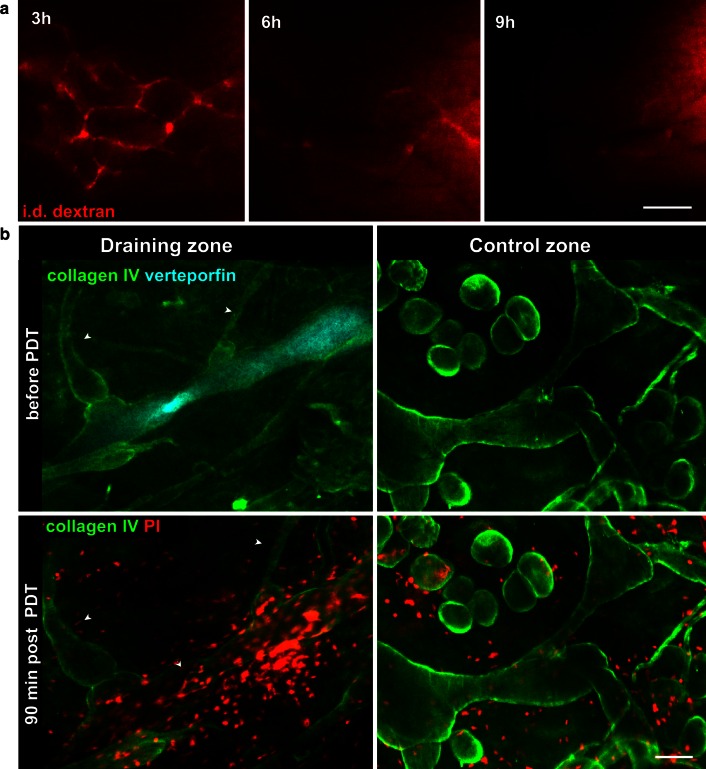
Lymphatic occlusion is delayed as compared to lymphatic endothelial cell death. **a** Lymphatic drainage did not become occluded until 6–9 h after PDT. **b** Intravital immunostaining of ear dermis with collagen IV antibody (*green*), verteporfin before PDT (*cyan*; *top*) and propidium iodide (PI, *red*; *bottom*) 1.5 h after PDT demonstrates large number of necrotic cells within verteporfin-draining lymphatic collectors, including nuclei located in the lymphatic valve (*arrow*) as well as peri-lymphatic cells (*left*). Few necrotic cells (PI-positive nuclei) in and around lymphatic vessels of non-draining control region (*right*). In addition, non-draining lymphatics (*arrowheads*) that were directly connected with verteporfin-draining vessels remained PI-negative. *Scale bars* in **a** and **b** correspond to −500 μm; in **c** to −100 μm

Fluorescence microlymphangiography revealed that lymphatic-specific PDT with 25 J/cm^2^ and 25 ng verteporfin led to lymphatic occlusion after 6–9 h in all mice tested (Fig. 3a), in contrast to blood-vessel-targeted PDT, in which irradiated, i.v.-administered verteporfin destroys local blood vessels within 3 h [23]. In both lymphatic and blood vessel-specific PDT, vessels carrying the photosensitizer are occluded when irradiated; thus, the discrepancy between the timing for blood versus lymphatic vessel occlusion may reflect different mechanisms of vessel blockade rather than administration routes of photosensitizer.

In order to determine the mechanism of PDT-dependent toxicity, the dorsal ear dermis was live-immunostained for collagen IV and propidium iodide (PI), which labels nuclei of necrotic cells (Fig. 3b). Collecting lymphatics were identified morphologically by their bulbous shapes, varying diameters, and characteristic asymmetric (pre-collectors) or bicuspid valves (collectors) located at branch points with sparse pericyte coverage as deduced from their basement membrane imprints (Supplementary Fig. 1). In addition, due to the thinness of the dorsal dermis it was possible to visualize the separate networks of nerves, lymphatics and blood vessels, which allowed us to readily identify lymphatic vessels from other structures. We further confirmed this morphological identification by co-staining with podoplanin, a lymphatic-specific marker in the skin [35].

We found that after only 1.5 h, a number of elongated nuclei within lymphatic vessels, including those near the valves, as well as peri-lymphatic cells, were necrotic (i.e., PI-positive). At the same time, cells associated with lymphatic vessels in non-draining (control) regions, including those that were directly connected to verteporfin-draining vessels, remained PI-negative (Fig. 3b). The early onset of the necrotic death of lymphatic endothelial cells or peri-lymphatic cells did not correlate with lymphatic occlusion occurring 6–9 h after PDT suggesting that at least initially intact lining of collecting lymphatic is not essential for collector function.

### Regeneration of muscle coverage but not endothelial lining correlates with recovery of lymphatic drainage

**Fig. 4:**
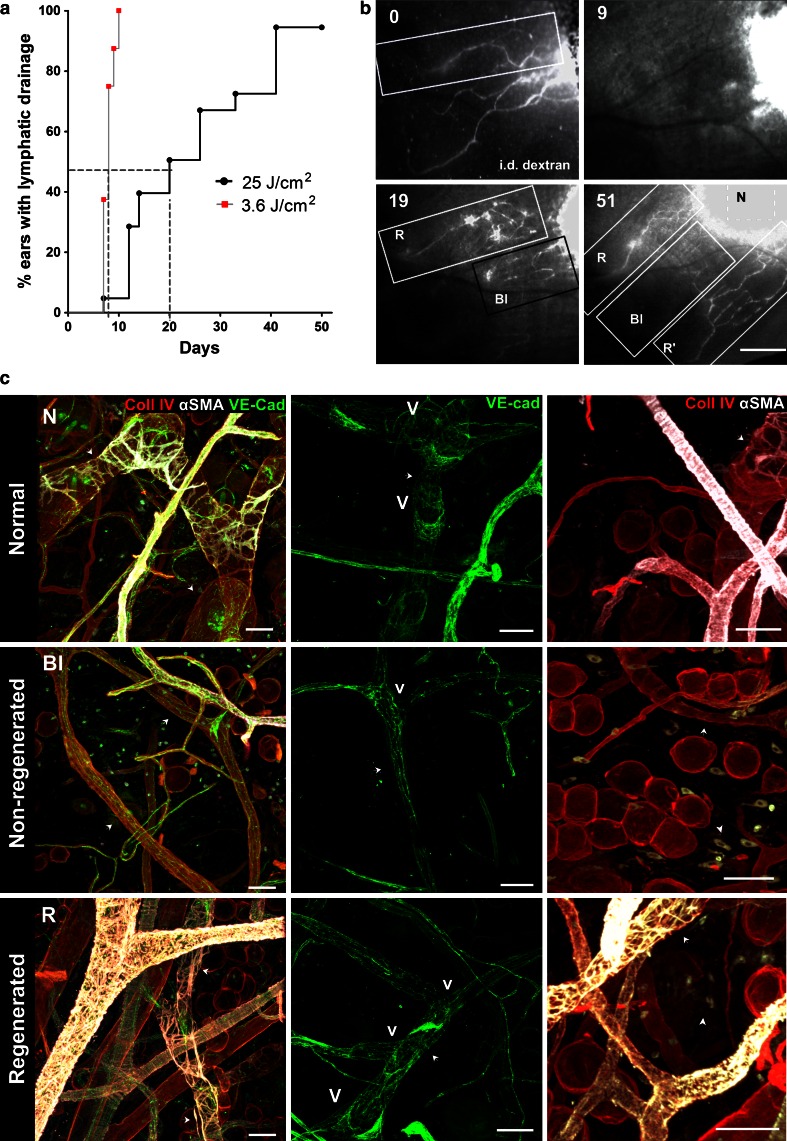
Kinetics of functional recovery and characteristics of regenerated lymphatics after PDT. **a** Kaplan-Mayer plot showing percent of ears with functional lymphatic drainage versus time for 3.6 versus 25 J/cm^2^ dose of light, with median recovery times of 8 and 20 days, respectively (*p* < 0.0001). The regeneration of lymphatic drainage was concluded when at least one collector drained TRITC-dextran injected in the region that was initially injected with verteporfin. **b** Lymphatic drainage recovery as detected by fluorescence microlymphangiography. Fluorescence microlymphangiography with intradermally (i.d.) injected TRITC-dextran performed on the same ear immediately after PDT (0) and again on days 9, 19, and 51 days after PDT. Three distinct skin zones could be identified: zone with normal (control) (N), blocked and non-regenerated (Bl), and functionally regenerated (*arrows*) (R). **c** Representative confocal images of lymphatic vessels in each zone 51 days post-PDT (the same mouse ear as in B and Supplementary Fig. 2) after whole-mount immunostaining for collagen IV (*red*), α-smooth muscle actin (αSMA, *white*) and VE-cadherin (*green*). *Left column* αSMA-positive pericytes that are normally sparse in control lymphatic vessels (N) are absent in non-regenerated lymphatics (Bl) but overabundant around regenerated lymphatic vessels (R). *Middle column* Valves (v) that normally exist at the lymphatic junctions (N) were absent at the junctions of non-regenerated lymphatics (Bl) and sparsely located at the junctions of regenerated collectors (R). *Right column* Myofibroblasts expressing αSMA that are lacking in normal tissue (N) are present in the tissue in areas with non-regenerated (Bl) and regenerated (R) vessels (*arrowheads*). *Arrows* indicate lymphatic vessels. *Bars* in **b** correspond to: 500 μm, in **c** to 50 μm

In order to investigate whether and how PDT-occluded lymphatic vessels can functionally regenerate, we followed lymphatic drainage by performing fluorescence microlymphangiography every 2–3 days over a period of 8 weeks (Fig. 4a). Lymphatic drainage was considered recovered when at least one collecting vessel could be observed draining the dextran from the site of injection to the base of the ear (Fig. 4b), even if some dysfunctional, PDT-occluded lymphatic vessels were still present in neighboring areas. Nevertheless, this definition of recovery was used for each ear because it implied that overall drainage was restored in the tissue. In 21 ears, the time to recovery of lymphatic function after PDT varied between 8 and 51 days, with median recovery times of 20 days for 25 J/cm^2^ and 8 days for 3.6 J/cm^2^ (*p* < 0.0001, Mantel-Cox test) (Fig. 4a).

Because fluorescence microlymphangiography combined with brightfield images allows specific collecting lymphatic vessels to be identified on different days by their relative position to major blood vessels (Fig. 4b and Supplementary Fig. 2a), we could compare the locations of regenerated versus original lymphatic vessels (Fig. 4b). Positions of vessels identified with fluorescence microlymphangiography could be also traced in fixed whole-mount ear preparations stained with vascular markers such as αSMA (Supplementary Fig. 2b–c). Ears were considered to have recovered lymphatic drainage function when at least one vessel could be seen draining the injected dextran. In these ears, we could also classify the major lymphatic vessels as either normal (N), which located in non-draining or control zones; blocked (Bl) or non-functional PDT-treated lymphatic vessels; or regenerated (R) which were initially blocked but functionally recovered (Fig. 4c, Supplementary Fig. 2b). Whole mount preparations were then stained for VE-cadherin (to identify endothelial cells), α-smooth muscle actin (SMA, to identify smooth muscle cells and myofibroblasts), and collagen IV (to identify basement membrane). In contrast to normal collecting vessels with sparse SMC coverage (Fig. 4c, N), regenerated lymphatic collectors were wrapped with vein-like dense SMC support (Fig. 4c, R). Surprisingly, blocked or non-regenerated lymphatic collectors were almost completely lined with lymphatic endothelium (Fig. 4c, Bl), although they were completely deprived of SMCs and their average diameter was approximately half that of control and regenerated lymphatics. Non-vascular αSMA-positive cells (myofibroblasts) were also present in the tissue stroma surrounding the occluded and regenerated lymphatics throughout the regeneration time (arrowheads in Fig. 4c). This was not paralleled with the formation of highly vascular granulation tissue or intense tissue remodeling that normally is associated with myofibroblasts-driven tissue remodeling processes [36, 37].

## Discussion

Aberrant lymphangiogenesis has been associated with tumor metastasis [4, 38], transplant rejection [12–15, 39], and tumor immune tolerance [10, 40], and thus developing strategies to destroy pathological lymphatic vessels may be therapeutically useful. For example, in allogenic corneal transplant, which is the standard of care for keratopathy, blocking lymphatic drainage may reduce the probability of transplant rejection [15, 41]. In addition, therapeutic antibodies that block lymphangiogenesis can reduce tumor metastasis in mouse models [4, 38].

PDT has been used following intravenously administered photosensitizer as anti-vascular and anti-tumor therapies for many decades [42]. Photosensitizer, located in tumor cells or in the blood vessels, transfers its energy to oxygen upon irradiation, causing the formation of a toxic singlet state oxygen radical that reacts with cell membrane components, proteins, lipids and nucleic acids, leading to cell death. This, in turn, results in biphasic blood-flow stasis and vessel hyperpermeability [23]. Unlike VEGFR-3 blocking strategies, which can only prevent new lymphatic growth, PDT can also ablate pre-existing lymphatic vessels. Recently, its potential for destroying peri-tumoral lymphatics and in-transit tumor cells was demonstrated by Tammela et al. [31]. Other potential therapeutic uses of lymphatic-specific PDT might include inhibition of spread of lymphatic-trafficking parasites or pathogens as well as slowing the clearance of locally delivered drugs [43, 44]. However, the ability to selectively destroy lymphatic vessels without also destroying local blood vessels has not been established.

We demonstrated here that local and specific ablation of dermal lymphatic vessels in mouse skin could be achieved with low doses of locally (intradermally) administered verteporfin and light, and that light dose can be tuned to modulate the time of lymphatic recovery. For example, low light doses of 3.5 J/cm^2^ could block lymphatic drainage for approximately 1 week, while 25 J/cm^2^ could prolong lymphatic recovery time to 3 weeks without adversely affecting blood vessel perfusion. This indicates that the higher light fluence was more effective in killing LECs and perilymphatic SMCs without augmenting the toxic bystander effects of PDT on blood vessels. Such flexibility is not only useful for a wide range of lymphatic physiology studies, but also e.g., for exploring the roles of lymphatic transport in immunity, since leukocyte trafficking from the periphery to the lymph nodes depends on intact lymphatic drainage [3].

We also found that varying doses of intradermally administrated verteporfin had distinct effects on blood versus lymphatic vessel occlusion. The use of higher photosensitizer doses (here, 100 ng) that target both blood and lymphatic vessels might be advantageous for example before cornea keratoplasty [13], where the simultaneous destruction of both vessels are desired. The lower doses of verteporfin (25 ng and below) could specifically block lymphatic drainage while leaving the blood vasculature functional.

In addition, we observed the recruitment and persistence of myofibroblasts, in the irradiated tissue throughout the period of lymphatic remodeling. Myofibroblasts, cells characteristic for tissue healing [45] were present in the tissue even though the tissue injury was limited to the lymphatic endothelial cells of collecting lymphatics and their SMCs coverage. Interestingly, only SMC-covered regenerated lymphatic vessels were functional, and their SMC coverage was notably denser than around normal vessels. In contrast, SMCs were completely absent in non-recovered lymphatic vessels, indicating that SMC recruitment was a necessary step in functional lymphatic regeneration. The higher SMC coverage around regenerated lymphatic vessels correlated with smaller vessel diameters, potentially suggesting that the smaller diameters were due to increased contractile forces from higher SMC coverage. Dense pericyte coverage of regenerating lymphatic vessels might also help stabilize lymphatic vessels similar to their effects during sprouting angiogenesis [46].

In conclusion, PDT can be used to selectively ablate lymphatic vessels and in turn block lymphatic drainage in a specified region. By modulating light fluence and photosensitizer dose, one can control the specificity of PDT to local lymphatic vessels versus blood vessels, as well as control the time for lymphatic drainage to be restored. Mechanisms of lymphatic occlusion and restoration need further investigation, but our studies suggest that PDT-blocked vessels become re-populated with LECs and pericytes before drainage is restored.

## Electronic supplementary material

Below is the link to the electronic supplementary material.

ESM1 JPEG (1.986KB)

ESM2 JPEG (1.191KB)

## Abbreviations

BM: Basement membrane
PDT: Photodynamic therapy
PI: Propidium iodide
SMC: Smooth muscle cell
TRITC-dextran: Tetramethyl rhodamine iso-thiocyanate-dextran
