# Supplementary figures and images for: Optimization and regeneration kinetics of lymphatic-specific photodynamic therapy in the mouse dermis
Source: Angiogenesis. 2013 Jul 28;17(2):347–57. doi: 10.1007/s10456-013-9365-6 (PMC3978193; doi:10.1007/s10456-013-9365-6)

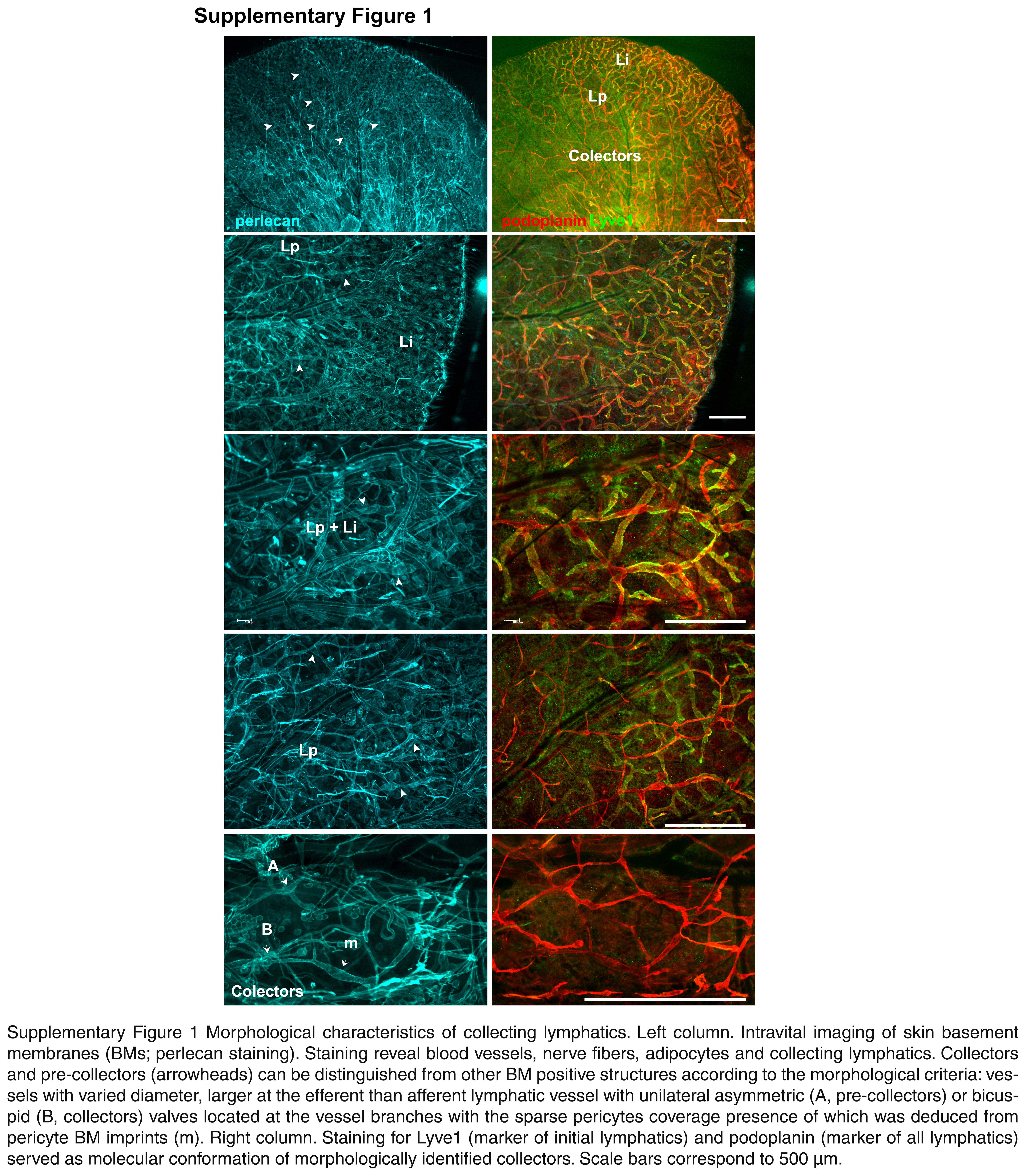

Supplement: Supplementary file 1 — ESM1 JPEG (1.986KB) [file 10456_2013_9365_MOESM1_ESM.jpg]

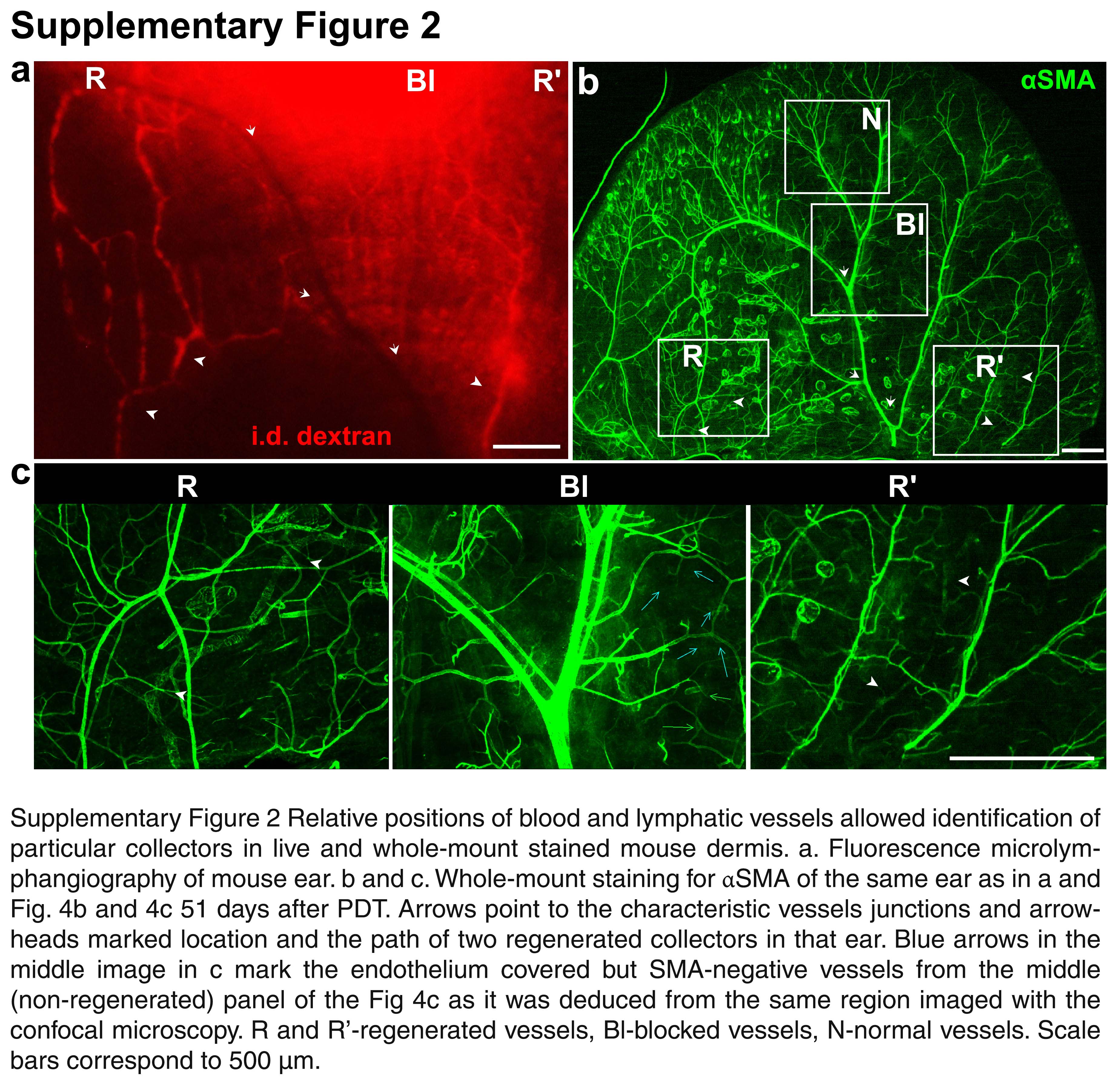

Supplement: Supplementary file 2 — ESM2 JPEG (1.191KB) [file 10456_2013_9365_MOESM2_ESM.jpg]
